# Large-scale multi-omics enhance risk prediction for type 2 diabetes

**DOI:** 10.1186/s12933-026-03223-y

**Published:** 2026-05-28

**Authors:** Ruijie Xie, Christian Herder, Ben Schöttker

**Affiliations:** 1 Division of Clinical Epidemiology of Early Cancer Detection, German Cancer Research Center, Im Neuenheimer Feld 581, 69120 Heidelberg, Germany; 2 Faculty of Medicine, Heidelberg University, 69115 Heidelberg, Germany; 3 Institute for Clinical Diabetology, German Diabetes Center (DDZ), Leibniz Center for Diabetes Research at Heinrich Heine University Düsseldorf, Düsseldorf, Germany; 4 German Center for Diabetes Research (DZD), Partner Düsseldorf, Munich- Neuherberg, Germany; 5 Department of Endocrinology and Diabetology, Medical Faculty, University Hospital Düsseldorf, Heinrich Heine University Düsseldorf, Düsseldorf, Germany

**Keywords:** Multi-omics, Type 2 diabetes, Prediction model, Biomarker, Cardiometabolic disease

## Abstract

**Background:**

Polygenic risk scores (PRS), metabolomics, and proteomics have each shown promise in improving type 2 diabetes risk prediction, but their combined utility beyond established clinical models remains unclear. We aimed to evaluate whether integrating multi-omics biomarkers enhances 10-year type 2 diabetes risk prediction beyond single-omics extensions and the clinical Cambridge Diabetes Risk Score (CDRS), which includes HbA_1c_ measurements.

**Methods:**

We analysed data from 42,840 UK Biobank participants without diagnosed diabetes at baseline. The study population was split into a derivation set (Phase 1 metabolomics release, *N* = 23,108) to fit models and an independent validation set (Phase 2 release, *N* = 19,732) to evaluate performance. Data for a PRS for type 2 diabetes, 11 metabolites, and 15 proteins were added to the CDRS to develop multi-omics prediction models. Model performance was evaluated using Harrell’s C-index and the net reclassification index (NRI).

**Results:**

During 10 years of follow-up, 1090 participants developed incident type 2 diabetes. Among individual omics layers, proteomics contributed the greatest improvement in predictive performance, increasing the C-index from 0.862 (clinical CDRS) to 0.884 (ΔC-index; + 0.022; *P* < 0.001), with a continuous NRI of 42.0%. The full multi-omics model further significantly increased the C-index compared to a model combining the clinical CDRS with proteomics data (C-index, 0.891; ΔC-index; + 0.007; *P* < 0.001).

**Conclusion:**

Integrating proteomics, metabolomics, and a diabetes-PRS into a clinical model substantially improves type 2 diabetes risk prediction beyond single-omics extensions. Several of the selected proteins and metabolites are on cardiovascular disease pathways, highlighting the link between diabetes and cardiovascular risk. However, the C-index difference between the proteomics extended and full multi-omics extended models is small, and the clinical models extended with proteomics data would be easier to translate into routine care because it needs only the measurement of 15 proteins. External validation and cost-effectiveness analyses are needed to support clinical adoption.

**Graphical abstract:**

**Supplementary Information:**

The online version contains supplementary material available at 10.1186/s12933-026-03223-y.

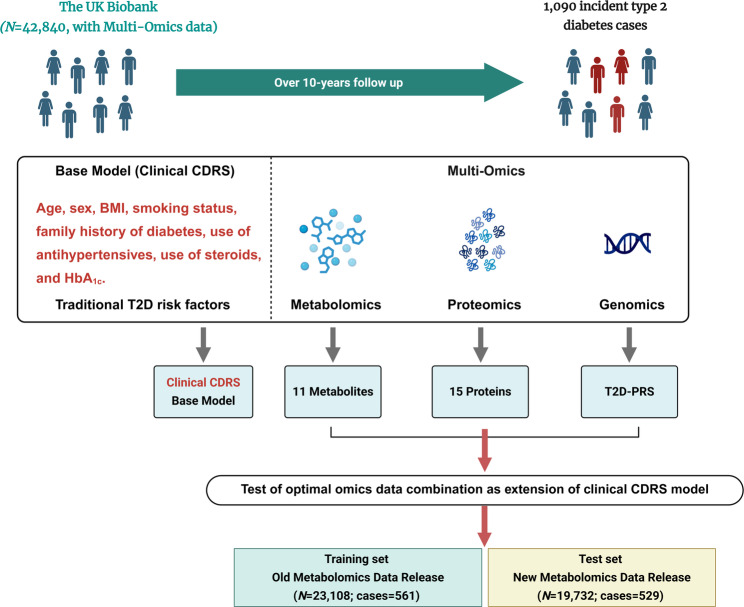

**Research insights**

**Table:** 

**What is currently known about this topic?**	Early type 2 diabetes (T2D) risk identification is crucial for effective prevention
	T2D prevention is also helpful for cardiovascular event prevention, as these can follow T2D.
	Clinical risk scores predict T2D well but there is still room for improvement.
**What is the key research question?**	Can multi-omics data enhance T2D risk prediction beyond clinical models?
**What is new?**	Multi-omics, especially proteomics, significantly improve T2D risk prediction beyond clinical models. Identified biomarkers link T2D risk to cardiometabolic pathways.
	A 15-protein panel offers a pragmatic, scalable prediction tool.
**How might this study influence clinical practice?**	Using the 15-protein extended clinical risk model could improve T2D risk assessment and prevention.

## Introduction

Type 2 diabetes is a major global health concern, with its rising prevalence leading to increased morbidity, premature mortality, and substantial economic burden [1–3]. Early identification of individuals at high risk is essential for implementing targeted preventive strategies to delay or prevent disease onset [4, 5]. However, existing prediction models often lack specificity and fail to capture the complex interplay of biological and environmental factors contributing to type 2 diabetes development, thereby limiting their clinical utility [6, 7].

To address these limitations, genomics, metabolomics, and proteomics have been increasingly explored as complementary tools for risk prediction [8–10]. Genome-wide association studies (GWAS) have identified numerous genetic variants associated with type 2 diabetes, while metabolomics and proteomics have uncovered key pathways involved in insulin resistance, inflammation, and metabolic dysfunction [11–13]. However, polygenic risk scores (PRS) have generally shown limited added value over traditional clinical models, and although metabolomics and proteomics have each demonstrated promising results, their combined predictive utility remains insufficiently explored [14–17]. To date, only one study has systematically assessed the integration of genomics, metabolomics, and proteomics for type 2 diabetes prediction in a multi-omics approach [18]. In the EPIC-Norfolk study (*N* = 1105), Carrasco-Zanini et al. reported that integrating the top 10 features from genomics, metabolomics, and proteomics significantly improved 10-year type 2 diabetes prediction over a clinical model alone (C-index: 0.82 vs. 0.87) [18]. However, large-scale, population-based studies are still lacking to assess the comparative and joint predictive value of different omics layers, and to identify the optimal omics combination for improving type 2 diabetes risk prediction.

In previous research using the UK Biobank (UKB), our group developed proteomics-based and metabolomics-based type 2 diabetes risk models that significantly improved the predictive performance of the clinical Cambridge Diabetes Risk Score (CDRS) [19, 20]. The CDRS is a sex-independent model that incorporates age, sex, body mass index (BMI), family history of diabetes, smoking status, and use of antihypertensive and steroid medications, with optional inclusion of glycated hemoglobin (HbA_1c_) [18, 21]. Separately, the UKB has also released a genome-wide type 2 diabetes polygenic risk score (T2D-PRS).

We aim to build upon this previous research on the single omics layers and combine the selected features from proteomics [19], metabolomics [20], and genomics [22] with the clinical CDRS model [18, 21] to determine the optimal combination of omics layers for 10-year type 2 diabetes risk prediction.

## Methods

### Study population

The UKB is a large prospective cohort comprising 502,414 participants aged 37 to 73 years, recruited from 13 March 2006 to 1 October 2010 across 22 assessment centers in England, Scotland, and Wales [23]. The cohort includes extensive phenotypic and genetic data, encompassing blood and urine biomarkers, whole-body imaging, lifestyle factors, anthropometric measurements, and genomic sequencing. UK Biobank received ethical approval from the North West Multi-centre Research Ethics Committee (REC reference 11/NW/0382). This study was conducted under UK Biobank application number [101633].

The starting point for the current analysis was a subsample of 52,995 participants with available proteomics measurements (Supplemental Fig. S1). We excluded participants who lacked polygenic risk score data (*n* = 552) or metabolomics data (*n* = 447), resulting in 51,996 participants with complete multi-omics data. Participants with diagnosed, potentially undiagnosed (baseline HbA_1c_ ≥ 6.5% [48 mmol/mol] [24]) and unknown diabetes status of any type at baseline were excluded (*n* = 3,762). Diagnosed diabetes was determined using self-reported medical history, primary care records, hospital admission data, glucose-lowering medication in self-reported data or prescription records before baseline. Finally, we excluded participants who were not randomly selected for the proteomics measurements (*n* = 5,394). Ultimately, 42,840 participants were included in the final analysis.

To ensure robust model evaluation and address potential overfitting, the study population was split into two completely independent, non-overlapping cohorts based on the UKB NMR metabolomics data release phases. The derivation set comprised 23,108 participants from the first metabolomics release (Phase 1, released in March 2021, covering samples measured between June 2019 and April 2020). The validation set comprised 19,732 participants from the second metabolomics release (Phase 2, released in July 2023, covering samples measured between April 2020 and June 2022).

### Reference model: the clinical CDRS

The CDRS is a well-established tool for predicting future risk of type 2 diabetes. It incorporates age, sex, BMI, family history of diabetes, smoking status, and the use of antihypertensive and steroid medications [21]. In this study, we used the clinical version of the CDRS that additionally includes HbA_1c_ levels [18]. We fit each of the component risk factors of the clinical CDRS as independent variables in our derivation set to re-estimate the coefficients specifically for the UKB population. This recalibrated model is referred to as the “clinical CDRS model”. In the UKB, age, sex, family history of diabetes, and smoking status were collected through standardized questionnaires. Information on prescribed medications was obtained through self-reported data, verbal interviews, and linkage with primary care records. Anthropometric measurements, including weight and height, were performed by trained staff at assessment centers. HbA_1c_ levels were measured from whole-blood samples using high-performance liquid chromatography on the Variant II platform (Bio-Rad Laboratories) [25].

### Proteomics data

Proteomic profiling was conducted on EDTA-plasma samples collected at baseline, mostly in a non-fasting state. The assay protocols, including sample handling and selection procedures, have been described previously [26]. Briefly, Olink Proteomics applies the proximity extension assay (PEA) technology, which uses pairs of antibodies linked to complementary oligonucleotides to detect target proteins [27–29]. A total of 2,923 unique proteins were measured using the Olink Explore 3072 platform (Olink Proteomics, Uppsala, Sweden). As outlined in our prior study [19], proteins with more than 20% missing values or over 25% of values below the detection limit were excluded, resulting in a final panel of 2,085 proteins for biomarker selection. In our previous UKB data analysis [19], we applied least absolute shrinkage and selection operator (LASSO) regression with bootstrap resampling to identify a parsimonious set of 15 proteins with high predictive value for type 2 diabetes (Supplemental Table S1). Incorporating these proteins into the clinical CDRS model significantly improved risk discrimination, with the C-index increasing from 0.831 to 0.860 (*P* < 0.001). In the present analysis, we evaluated only these 15 preselected proteins.

### Metabolomic data

Plasma metabolomic profiling was conducted on EDTA samples, mostly collected in a non-fasting state, using a high-throughput nuclear magnetic resonance (NMR) platform developed by Nightingale Health [30]. This platform quantified 249 circulating metabolites, encompassing a broad spectrum of molecular classes such as lipids, fatty acids, amino acids, ketone bodies, and other low-molecular-weight compounds. Among these, 168 metabolites were measured in absolute concentrations, including 61 composite biomarkers derived from 107 directly quantified metabolites. The remaining 81 metabolites were expressed as concentration ratios. In our previous UKB data analysis [20], we applied LASSO regression with bootstrap resampling to identify a parsimonious set of 11 metabolites with strong predictive value (Supplemental Table S1). Incorporation of these metabolites into the clinical CDRS model significantly improved risk discrimination, with the C-index increasing from 0.815 to 0.834 (*P* < 0.001). In the present analysis, only these 11 preselected metabolites were evaluated.

### Polygenic risk score for type 2 diabetes

The PRS for type 2 diabetes was provided by the UK Biobank PRS Release [22]. This score was derived using a Bayesian modelling approach, trained on meta-analysed summary statistics from 5 external GWAS datasets, and subsequently optimized using internal UK Biobank data. In internal benchmarking analyses, the UKB T2D-PRS demonstrated strong predictive performance and was shown to be comparable or superior to previously published PRSs (e.g., Mahajan et al. and Khera et al.) [22, 31, 32].

### Type 2 diabetes ascertainment

 Participants were censored at the earliest of the following events: diagnosis of type 2 diabetes, death, loss to follow-up, or completion of the 10-year follow-up period. Incident type 2 diabetes was identified using three complementary data sources in the UKB [33]. The detailed diagnosis and medication codes are provided in Supplemental Table S2. To minimize false positives from off-label medication use (e.g., metformin for polycystic ovary syndrome), glucose-lowering medication (ATC code A10) alone was insufficient for diagnosis; it had to be corroborated by a diagnostic code or HbA_1c_ ≥ 6.5%.

### Statistical analyses

### General remarks

All statistical analyses were conducted in R software (version 4.4.0, R Foundation for Statistical Computing, Vienna, Austria). Statistical significance was set at *P* < 0.05 (two-sided). Missing values of variables of the clinical CDRS (variable with the highest proportion of missing values was HbA_1c_ with 4.8%) and multi-omics (mostly complete with a few proteins with up to 20% of missing values) were single imputed using the chained equations method with random forest algorithms implemented in the R package *missForest* (version 1.5) [34].

### Test of model performance

The study design is illustrated in the graphical abstract. Panels of 15 proteins, 11 metabolites, and the UK Biobank’s T2D-PRS, derived in previously published selection procedures [19, 20, 22], were combined with the variables of the clinical CDRS to develop a 10-year type 2 diabetes risk prediction model [21]. Biomarker selection and model training were conducted in the derivation set, and model performance was evaluated in the independent validation set (Fig. S1).

Model discrimination was evaluated using Harrell’s C-index. Differences in C-index between models were assessed using the method by Kang et al. for comparing correlated C-indices in survival analysis, implemented in the R package *compareC* (version 1.3.2) [35]. The incremental C-statistic of each selected biomarker was also estimated. In a first step, we assessed the predictive performance of each individual omics layer when added to the clinical CDRS model. In a second step, the combination of the best-performing omics layer and the clinical CDRS was selected as the reference model, and we tested whether adding more omics layers improved its predictive ability.

Risk reclassification was evaluated using the continuous net reclassification index (NRI) and the integrated discrimination index (IDI) [36, 37]. The proportional hazards assumption was tested using Schoenfeld residuals, and no violations were detected. Model calibration was assessed by plotting observed 10-year type 2 diabetes incidence against predicted risks across deciles of absolute risk in the validation set.

We additionally conducted subgroup analyses stratified by age (< 55 and ≥ 55 years), BMI (< 25, 25–29.9, and ≥ 30 kg/m²), sex (male and female), and prediabetes status (no prediabetes [HbA1c < 5.7%; <39 mmol/mol] and prediabetes [HbA_1c_ 5.7–6.4%; 39–47 mmol/mol]) according to the ADA criteria [24].

Most blood samples were collected in a non-fasting state. In a sensitivity analysis, we checked if adding self-reported fasting time prior blood sampling (continuous in hours) to the metabolomics extended reference model changes its predictive performance (especially of glucose). To put the model performance of the multi-omics extended into context, we compared them also to a model extending the clinical CDRS with routinely collected cardiometabolic biomarkers available in the UK Biobank, including systolic and diastolic blood pressure, liver enzymes (alanine aminotransferase [ALT], aspartate aminotransferase [AST], gamma-glutamyltransferase [GGT]), C-reactive protein (CRP), lipids (total cholesterol, high-density lipoprotein [HDL] cholesterol, low-density lipoprotein [LDL] cholesterol, triglycerides), blood glucose, and creatinine.

### Associations of selected biomarkers with incident type 2 diabetes

To report hazard ratios (HRs) and 95% confidence intervals (CIs) for the associations of the selected biomarkers (per one standard deviation [SD] increment) with 10-year incident type 2 diabetes, each biomarker was entered separately into Cox proportional hazards models in the validation set (R package *survival* (version 3.5-5)). All models were adjusted for the variables of the clinical CDRS.

### Correlation matrix

To assess the independence and interrelationships among the selected predictors, pairwise Spearman correlation coefficients were calculated between the clinical risk factors (age, sex, BMI, and HbA_1c_) and the selected multi-omics biomarkers in the validation set.

## Results

### Baseline characteristics and incident type 2 diabetes cases

Table 1 presents the baseline characteristics of 42,840 participants included in the study, of whom 1090 developed type 2 diabetes during the 10-year follow-up period. The mean age of the total cohort was 56.3 years, with 44.5% being male. The mean HbA_1c_ was 35.0 mmol/mol, the mean BMI was 27.1 kg/m², and 19.7% had a family history of diabetes (parent or sibling, or both). The baseline characteristics are additionally presented separately for the derivation set (*N* = 23,108) and the validation set (*N* = 19,732), and no clinically meaningful differences were observed.

**Table 1:** Baseline characteristics of participants from the UK Biobank

Baseline characteristics	Total (*N* = 42,840)	Derivation set (*N* = 23,108)	Validation set (*N* = 19,732)
Male sex, n (%)	19,076 (44.5)	10,259 (44.4)	8817 (44.7)
Age (years), mean (SD)	56.3 (8.2)	56.4 (8.1)	56.3 (8.2)
HbA_1c_ (mmol/mol), mean (SD)	35.0 (3.7)	35.0 (3.7)	35.1 (3.6)
BMI (kg/m²), mean (SD)	27.1 (4.6)	27.1 (4.5)	27.1 (4.6)
BMI category, n (%)			
<25 kg/m^2^	14,823 (34.6)	8002 (34.6)	6821 (34.6)
25–27.49 kg/m^2^	10,621 (24.8)	5726 (24.8)	4895 (24.8)
27.5–29.99 kg/m^2^	8042 (18.8)	4375 (18.9)	3667 (18.6)
≥30 kg/m^2^	9354 (21.8)	5005 (21.7)	4349 (22.0)
Cigarette smoking, n (%)			
Never	23,662 (55.2)	12,860 (55.7)	10,802 (54.7)
Ex-smoker	14,647 (34.2)	7860 (34.0)	6787 (34.4)
Current smoker	4531 (10.6)	2388 (10.3)	2143 (10.9)
Family history of diabetes, n (%)			
None	34,376 (80.2)	18,497 (80.0)	15,879 (80.5)
Parent or sibling	7511 (17.5)	4079 (17.7)	3432 (17.4)
Parent and sibling	953 (2.2)	532 (2.3)	421 (2.1)
Prescribed medication, n (%)			
Anti-hypertensive	7834 (18.3)	4228 (18.3)	3606 (18.3)
Steroid	289 (0.7)	168 (0.7)	121 (0.6)

BMI, body mass index; HbA_1c_, glycated hemoglobin; SD, standard deviation; T2D, type 2 diabetes.

Continuous variables are presented as mean (standard deviation) and compared using the independent t-test. Categorical variables are presented as counts (percentages) and compared using the chi-square test.

### Correlations between clinical and multi-omics features

**Fig. 1:**
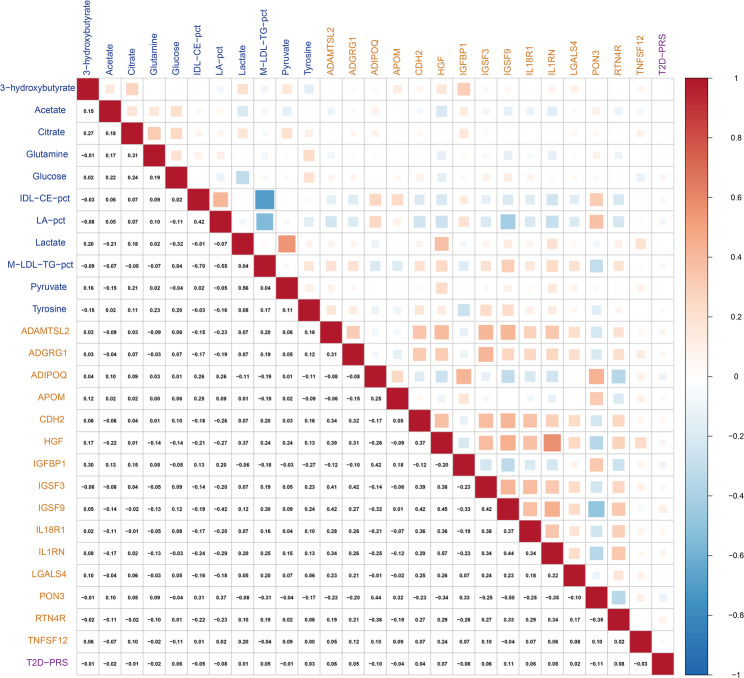
Spearman correlation matrix of the selected multi-omics biomarkers and the T2D-PRS in the validation set (*N* = 19,732) Metabolite names are shown in blue, protein names in orange, and the T2D-PRS in purple.* Abbreviations*: ADAMTSL2, A disintegrin and metalloproteinase with thrombospondin repeats-like 2; ADGRG1, Adhesion G-protein coupled receptor G1; ADIPOQ, Adiponectin; APOM, Apolipoprotein M; CDH2, Cadherin-2; HGF, Hepatocyte growth factor; IDL-CE-pct, cholesteryl esters to total lipids in IDL percentage; IGFBP1, Insulin-like growth factor-binding protein 1; IGSF3, Immunoglobulin superfamily member 3; IGSF9, Immunoglobulin superfamily member 9; IL18R1, Interleukin-18 receptor 1; IL1RN, Interleukin-1 receptor antagonist; LA-pct, linoleic acid to total fatty acids percentage; LGALS4, Galectin-4; M-LDL-TG-pct, triglycerides to total lipids in medium LDL percentage; PON3, Paraoxonase/lactonase 3; RTN4R, Reticulon-4 receptor; T2D-PRS, polygenic risk score for type 2 diabetes; TNFSF12, TNF-related weak inducer of apoptosis

Figure 1 illustrates the Spearman correlation matrix among the T2D-PRS, the 11 selected metabolites, and the 15 selected proteins in the validation set. Almost no correlation (all |r| ≤ 0.11) was observed between the T2D-PRS and biomarkers from the other omics layers. Correlations between metabolites and proteins were generally weak to moderate (all |r| < 0.5). Within individual omics layers, only one pair of biomarkers demonstrated a strong correlation (M-LDL-TG-pct and LA-pct: *r* = − 0.70), while a few others showed moderate correlations.

### Associations of selected omics biomarkers with type 2 diabetes

**Fig. 2:**
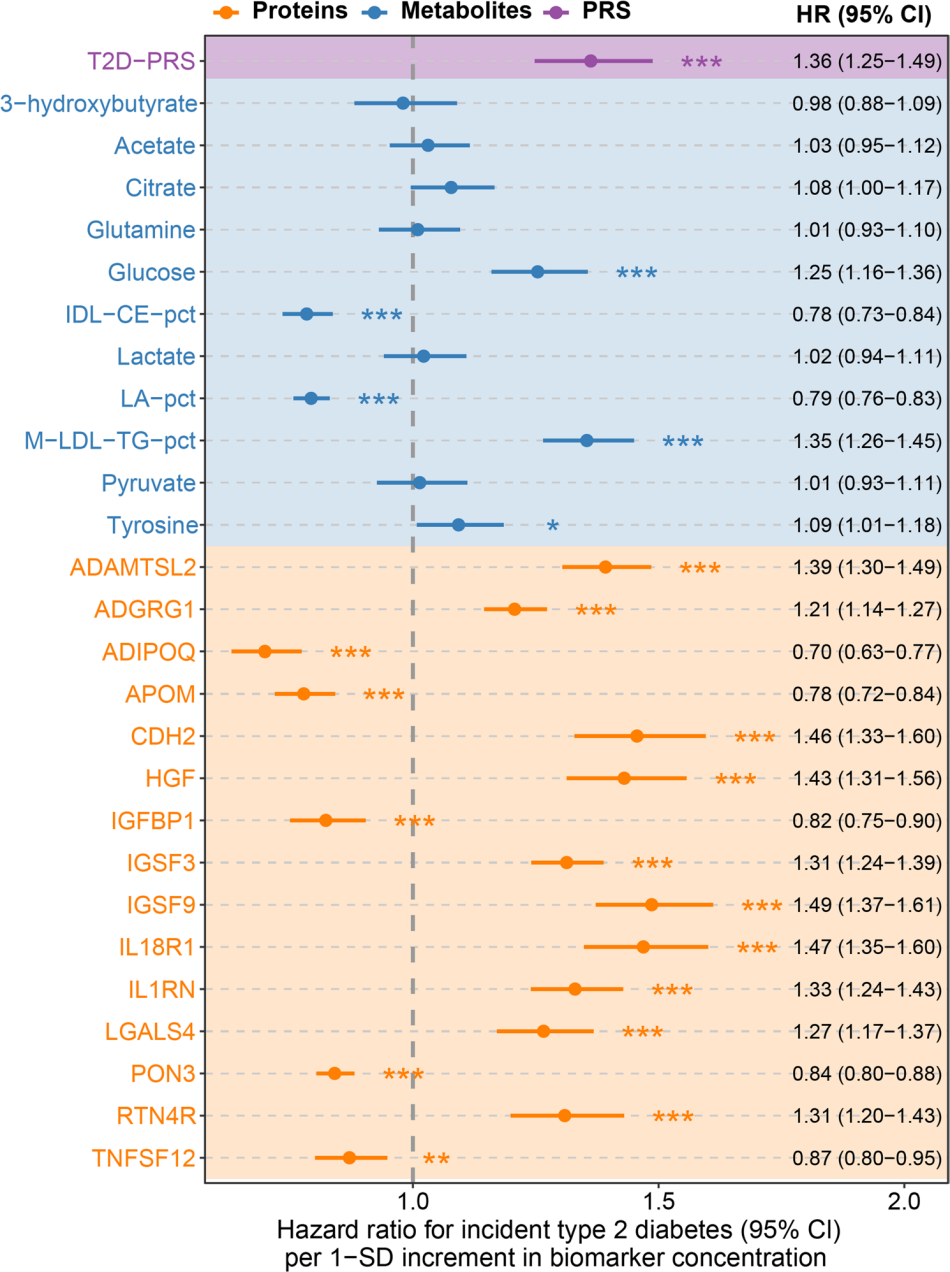
Associations of individual multi-omics biomarkers with incident type 2 diabetes in the validation set (*N* = 19,732) Hazard ratios and 95% confidence intervals for one standard deviation increment in biomarker concentration for type 2 diabetes incidence. Analyses were adjusted for all variables included in the clinical CDRS model. Asterisks indicate the level of statistical significance (*P < 0.05, **P < 0.01, ***P < 0.001).* Abbreviations*: ADAMTSL2, A disintegrin and metalloproteinase with thrombospondin repeats–like 2; ADGRG1, Adhesion G-protein coupled receptor G1; CDH2, Cadherin-2; CDRS, Cambridge Diabetes Risk Score; HGF, Hepatocyte growth factor; IDL-CE-pct, cholesteryl esters to total lipids in IDL percentage; IGFBP1, Insulin-like growth factor-binding protein 1; IGSF, Immunoglobulin superfamily member; IL1RN, Interleukin-1 receptor antagonist; IL18R1, Interleukin-18 receptor 1; LA-pct, linoleic acid to total fatty acids percentage; M-LDL-TG-pct, triglycerides to total lipids in medium LDL percentage; PON3, paraoxonase/lactonase 3; RTN4R, Reticulon-4 receptor; SD, standard deviation; T2D-PRS, polygenic risk score for type 2 diabetes; TNFSF12, TNF-related weak inducer of apoptosis

Figure 2 presents the associations of the selected multi-omics biomarkers with incident type 2 diabetes. Most biomarkers were significantly associated with type 2 diabetes risk, except for sixt metabolites that did not reach statistical significance. The T2D-PRS was statistically significantly associated with type 2 diabetes risk (HR per 1 SD: 1.36, 95% CI: 1.25–1.49). Among the metabolomic biomarkers, M-LDL-TG-pct (triglycerides to total lipids in medium LDL percentage) demonstrated the strongest association with T2D (HR per 1 SD: 1.35, 95% CI: 1.26–1.45) followed by glucose (HR per 1 SD: 1.25, 95% CI: 1.16–1.36). Among the proteomic biomarkers, IGSF9 (Immunoglobulin superfamily member 9) demonstrated the strongest association with type 2 diabetes (HR [95%CI] per 1 SD): 1.49 [1.37–1.61].

### Improvements in risk prediction by multi-omics integration

**Fig. 3:**
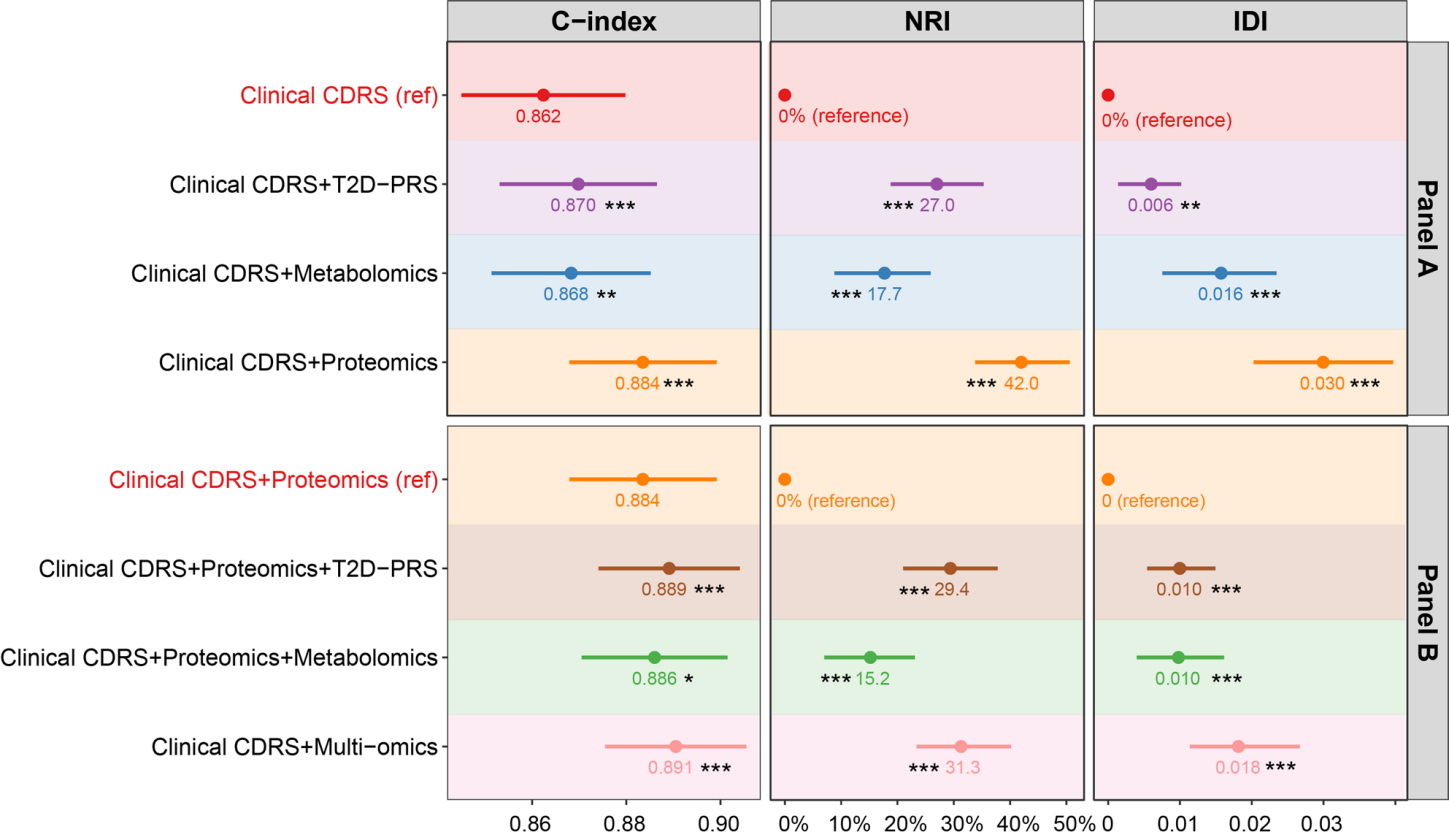
Predictive performance of the clinical CDRS model extended by multi-omics layers for 10-year type 2 diabetes risk prediction in the validation set (*N* = 19,732). Panel A compares the performance of the clinical CDRS model with its single-omics extensions, including the T2D-PRS, metabolomics, and proteomics. Panel B evaluates the incremental benefit of adding the T2D-PRS or metabolomics to the proteomics-extended model, as well as the full multi-omics model integrating all three layers. Model performance was assessed using C-index, continuous NRI, and IDI. In Panel A, the clinical CDRS served as the reference model. In Panel B, Clinical CDRS + Proteomics served as the reference. Asterisks indicate the statistical significance of performance differences compared with the respective reference model (*P < 0.05, **P < 0.01, ***P < 0.001). The multi-omics model refers to the integration of 15 selected proteins, 11 metabolites, and a T2D-PRS.* Abbreviations*:CDRS, Cambridge Diabetes Risk Score; T2D-PRS, polygenic risk score for type 2 diabetes; NRI, net reclassification index; IDI, integrated discrimination improvement

Figure 3 shows the predictive performance of the clinical CDRS model extended by the three multi-omics layers. The C-index of the clinical CDRS model was 0.862. Adding the T2D-PRS (ΔC-index = + 0.008; *P* < 0.001) or metabolomics data (ΔC-index = + 0.006; *P* = 0.008) resulted in modest improvements, while integrating proteomics data provided the most substantial improvement (ΔC-index = + 0.022; *P* < 0.001), underscored by an especially high continuous NRI (42.0%). Thus, in the next step, the proteomics-extended clinical CDRS model was chosen as the reference model. Adding either the T2D-PRS or metabolomics to the proteomics-extended model further improved discrimination slightly. Adding both the T2D-PRS and metabolomics to the proteomics-extended model enhanced discrimination the most (ΔC-index = + 0.007; *P* < 0.001).

The ß-coefficients of all variables of the main models (CDRS + Proteomics and CDRS + multi-omics) are detailed in Supplemental Table S3. Supplemental Fig. S2 presents the calibration curves for these models, indicating good agreement between predicted and observed event rates across all models.

A model extending the clinical CDRS with routinely collected cardiometabolic biomarkers had a C-index of 0.875, which outperformed the models adding only the T2D-PRS and only metabolomics, but did not perform better than the models adding only proteomics (C-index of 0.884) or multi-omics (C-index of 0.891).

**Fig. 4:**
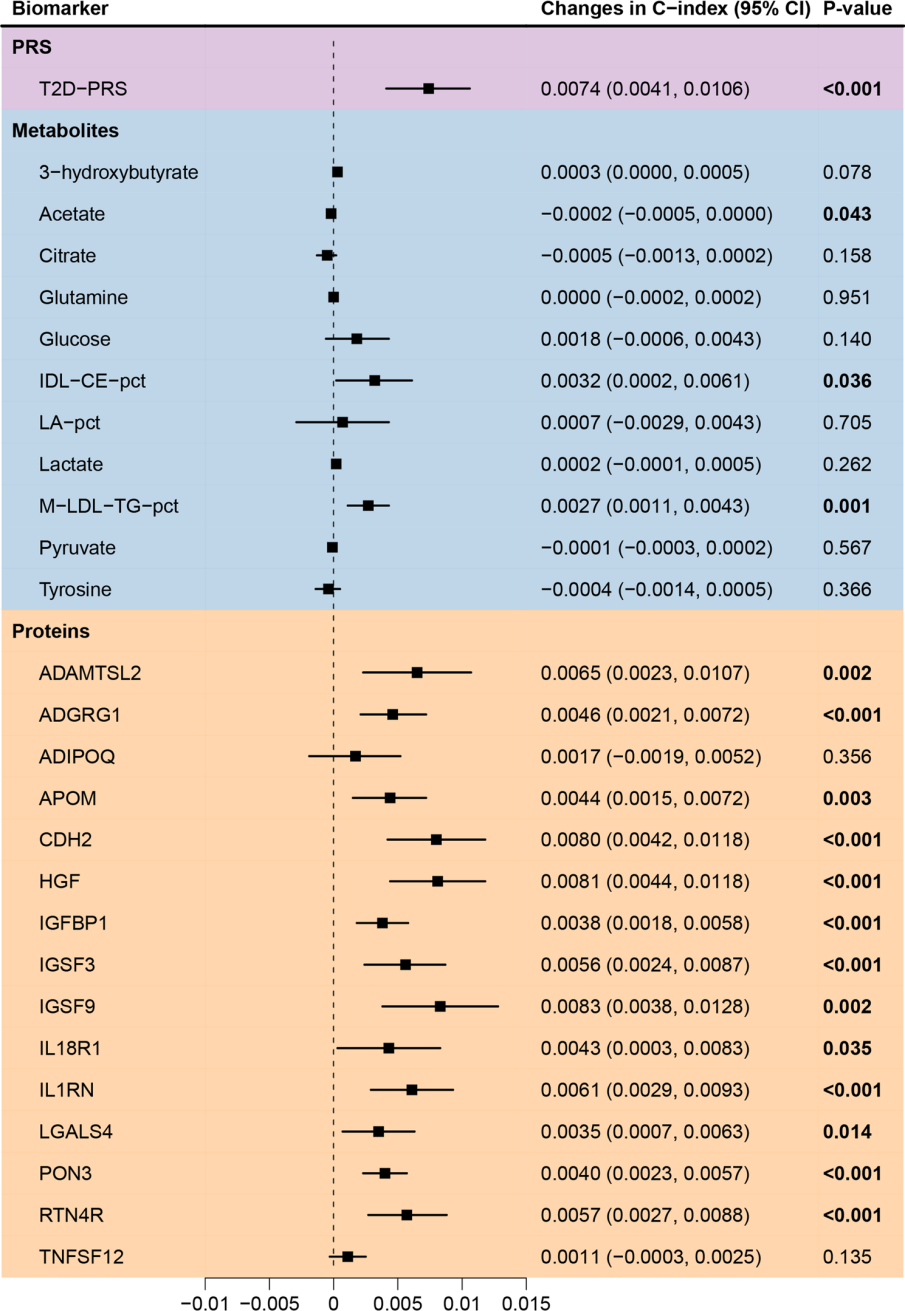
Incremental contributions of individual biomarkers to risk prediction. Forest plot displaying the incremental changes in the C-index when each biomarker is added to the clinical CDRS model.* Abbreviations*: ADAMTSL2, A disintegrin and metalloproteinase with thrombospondin repeats–like 2; ADGRG1, Adhesion G-protein coupled receptor G1; CDH2, Cadherin-2; CDRS, Cambridge Diabetes Risk Score; HGF, Hepatocyte growth factor; IDL-CE-pct, cholesteryl esters to total lipids in IDL percentage; IGFBP1, Insulin-like growth factor-binding protein 1; IGSF, Immunoglobulin superfamily member; IL1RN, Interleukin-1 receptor antagonist; IL18R1, Interleukin-18 receptor 1; LA-pct, linoleic acid to total fatty acids percentage; M-LDL-TG-pct, triglycerides to total lipids in medium LDL percentage; PON3, paraoxonase/lactonase 3; RTN4R, Reticulon-4 receptor; T2D-PRS, polygenic risk score for type 2 diabetes; TNFSF12, TNF-related weak inducer of apoptosis

Figure 4 displays the incremental C-index improvements of each selected biomarker of the multi-omics data when added individually to the clinical CDRS model. The T2D-PRS showed a significant contribution, increasing the C-index by 0.0074. Proteomic biomarkers contributed more to model performance than metabolites, with 13 demonstrating a statistically significant C-index increase, which was otherwise only observed for 3 metabolites. Among the proteins, IGSF9, HGF, and CDH2 provided the greatest improvements in predictive performance with increases in C-index ≥ 0.008. Among metabolites, M-LDL-TG-pct had the highest predictive contribution, increasing the C-index by 0.0027. Adjusting for self-reported fasting time did not alter the predictive performance of the metabolomics-extended model (C-index remained at 0.868) or the incremental C-index for glucose (remained at 0.0018).

Subgroup analyses, stratified by age, BMI, sex, and pre-diabetes status, showed a pattern consistent with the one observed for the total population: proteomics improved the predictive ability of the clinical CDRS more than the T2D-PRS and metabolomics alone, but the full multi-omics model had the highest C-index of all models (Supplemental Fig. S3). The only exception was observed among study participants with low-to-normal weight (BMI < 25 kg/m²), for whom all models performed equally. Notably, the highest C-index of 0.906 was observed for the full multi-omics model in the younger subgroup (< 55 years). For comparison, the C-index in the older subgroup (≥ 55 years) was 0.877. In the important T2D high-risk group with prediabetes, adding proteomics considerably increased the C-index from 0.761 (clinical CDRS) to 0.802.

## Discussion

This study investigated whether integrating multi-omics data into a clinical risk model could improve 10-year prediction of type 2 diabetes in a large, population-based cohort of 42,840 adults without previously diagnosed diabetes. We combined a T2D-PRS, 11 metabolites, and 15 proteins with the clinical CDRS and observed significant gains in predictive performance in terms of model discrimination and reclassification. While the full multi-omics model achieved the highest discrimination, its incremental benefit over the proteomics-extended model was modest.

To our knowledge, this is the first large-scale study to systematically evaluate whether integrating genomics, metabolomics, and proteomics improves type 2 diabetes risk prediction beyond single-omics extensions and established clinical models. Previous studies have primarily examined the predictive value of individual omics layers [13, 38–40]. T2D-PRSs have been investigated as early predictors of type 2 diabetes, but their incremental value beyond clinical models has generally been limited [14, 41]. Metabolomics and proteomics have revealed biological pathways relevant to insulin resistance and metabolic dysfunction, and several studies have demonstrated considerable abilities in diabetes risk prediction for these omics data [13, 16, 33, 42–46]. However, these studies focused on single omics layers and did not assess their joint predictive value. In the only prior multi-omics study, conducted in the EPIC-Norfolk cohort (*N* = 1,105; 375 incident cases), Carrasco-Zanini et al. reported that integration of top features from genomics, proteomics, and metabolomics significantly improved type 2 diabetes prediction compared to the clinical CDRS model (C-index: 0.82 to 0.87; *P* = 0.045), whereas single-omics models showed no significant benefit [18]. Our findings confirm and extend these results in a substantially larger cohort (*N* = 42,840; 1,090 incident cases), demonstrating that multi-omics integration significantly enhanced risk prediction (C-index: 0.862 to 0.891). The larger sample size of our cohort allowed more in-depth analyses and elucidated that each omics layer alone significantly improved the predictive abilities of the clinical CDRS, and that proteomics data contributed the largest C-index increase.

To contextualize the clinical relevance of these improvements, the addition of HDL cholesterol to the Framingham Risk Score typically yields a ΔC-index of ~ 0.01, which is widely considered clinically meaningful [47]. The improvement provided by our proteomics panel (ΔC-index = + 0.022) is substantially larger than this benchmark. The proteomics panel also outperformed a model of routinely available biomarkers, such as blood pressure, liver enzymes, CRP, lipids, blood glucose, and creatinine. Moreover, the substantial continuous NRI of 42.0% for the proteomics model indicates that a large proportion of individuals were more accurately classified into higher or lower risk trajectories.

This superior performance of the proteomics layer may reflect the fact that circulating proteins often act as downstream effectors of disease processes, more closely representing real-time pathophysiology. Notably, several of the identified biomarkers in our 15-protein panel, such as IL18R1, IL1RN, and HGF, as well as metabolites like M-LDL-TG-pct, are linked to established cardiometabolic pathways involved in inflammation and vascular health [48–52]. Our study identified IGSF9 as the strongest individual proteomic predictor of incident type 2 diabetes. While originally characterized for its role in the nervous system [53], emerging evidence strongly implicates IGSF9 in metabolic and inflammatory disease processes. Recent studies have identified IGSF9 as a top differentially expressed protein associated with both liver steatosis and fibrosis [54], and demonstrated its inverse association with insulin sensitivity and positive association with glycemic worsening [55]. Mechanistically, IGSF9 has been shown to regulate the complement system and liver inflammation [56], and modulate FAK/AKT signaling pathways [57].

Beyond improving overall prediction, multi-omics integration may help identifying individuals who fall below current clinical thresholds for intervention. Existing guidelines recommend preventive action for individuals classified as having prediabetes, defined as HbA_1c_ levels ≥ 5.7% (≥ 39 mmol/mol) according to ADA criteria [24]. However, this threshold may fail to identify all individuals at elevated risk for type 2 diabetes and its complications. Our results demonstrate that especially among participants without prediabetes (HbA_1c_ <5.7% [< 39 mmol/mol]), the multi-omics model significantly improved risk prediction compared to the clinical CDRS. Thus, multi-omics data may allow early identification of high-risk individuals before prediabetes is clinically detectable. This early identification is crucial, as it offers a window of opportunity for interventions aimed not only at preventing the onset of T2D but also at supporting earlier cardiometabolic prevention, including cardiovascular risk management [58].

Recent studies in translational omics have similarly emphasized the need for simplified, interpretable biomarker sets to enable real-world application [59, 60]. While the full multi-omics model achieved the highest overall performance in the total population as well as in people with or without prediabetes, adding proteomics alone achieved almost as good C-index increases in all populations. Thus, the proteomics-extended clinical CDRS model may represent a pragmatic balance between accuracy and feasibility for clinical translation considering the high costs and operational complexity of measuring multi-omics data in clinical settings. In fact, the plasma concentration measurements of only 15 proteins would be needed for the suggested model in this study. The OLINK Focus platform enables simultaneous measurement of up to 21 selected proteins from the broad OLINK Explore library with around 3000 proteins (https://olink.com/products/olink-focus). A developed 15-protein-panel could offer a cost-effective and scalable solution for implementation in clinical workflows.

This study has several strengths. This is the largest population-based study to evaluate the integration of proteomics, metabolomics, and a T2D-PRS into an established clinical model for type 2 diabetes risk prediction. The large sample size allowed testing each omics layer separately and to conduct a sub-group analysis by prediabetes status. Nonetheless, several limitations should be considered. First, the metabolomics data were derived from a targeted nuclear magnetic resonance platform limited to 250 metabolites, which may not capture the full metabolic complexity relevant to type 2 diabetes. Second, our analyses cannot be generalized to other populations than those with European ancestry aged 37–73 years. Third, while we utilized a rigorous internal validation strategy using completely independent, non-overlapping subsets of the UKB, external validation in independent cohorts with diverse ancestral backgrounds is essential before these multi-omics models can be widely adopted in routine clinical care. Currently, there is a lack of publicly available external cohorts with comparable large-scale, concurrent multi-omics data. Fourth, although we provide regression coefficients for the selected biomarkers, these may require recalibration when applied to other populations or measurement platforms.

## Conclusions

This study demonstrates for the first time in a large population-based cohort that integrating proteomics, metabolomics, and a T2D-PRS into an established clinical model significantly improves 10-year type 2 diabetes risk prediction beyond single-omics approaches. While the full multi-omics model yielded the highest overall accuracy, the proteomics-extended model alone achieved almost as good model improvements and may represent a more pragmatic option for clinical implementation. These findings highlight the potential of proteomics-based risk models to improve population-level diabetes risk stratification.

## Supplementary Information

Below is the link to the electronic supplementary material.

Supplementary Material 1.

## Data Availability

Data from the UK Biobank are available to bona fide researchers upon application through the UK Biobank Access Management System (https://www.ukbiobank.ac.uk/enable-your-research/apply-for-access).

## Abbreviations

ADA: American diabetes association
ALT: Alanine aminotransferase
AST: Aspartate aminotransferase
ATC: Anatomical therapeutic chemical
BMI: Body mass index
CDRS: Cambridge diabetes risk score
CI: Confidence interval
CRP: C-reactive protein
GGT: Gamma-glutamyltransferase
GWAS: Genome-wide association study
HDL: High-density lipoprotein
HR: Hazard ratio
IDI: Integrated discrimination improvement
IDL-CE-pct: Cholesteryl esters to total lipids in IDL percentage
IGSF9: Immunoglobulin superfamily member 9
LASSO: Least absolute shrinkage and selection operator
LDL: Low-density lipoprotein
M-LDL-TG-pct: Triglycerides to total lipids in medium LDL percentage
NMR: Nuclear magnetic resonance
NRI: Net reclassification index
PRS: Polygenic risk score
T2D-PRS: Type 2 diabetes polygenic risk score
UKB: UK Biobank

## References

[1] Global regional, national burden of diabetes. from 1990 to 2021, with projections of prevalence to 2050: a systematic analysis for the Global Burden of Disease Study 2021. Lancet. 2023;402(10397):203–34. 37356446 10.1016/S0140-6736(23)01301-6 PMC10364581

[2] Ahmad E, Lim S, Lamptey R, Webb DR, Davies MJ. Type 2 diabetes. Lancet. 2022;400(10365):1803–20. 36332637 10.1016/S0140-6736(22)01655-5

[3] Tomic D, Shaw JE, Magliano DJ. The burden and risks of emerging complications of diabetes mellitus. Nat Rev Endocrinol. 2022;18(9):525–39. 35668219 10.1038/s41574-022-00690-7 PMC9169030

[4] Group DPPR. Reduction in the incidence of type 2 diabetes with lifestyle intervention or metformin. N Engl J Med. 2002;346(6):393–403. 11832527 10.1056/NEJMoa012512 PMC1370926

[5] Tuomilehto J, Lindström J, Eriksson JG, Valle TT, Hämäläinen H, Ilanne-Parikka P, Keinänen-Kiukaanniemi S, Laakso M, Louheranta A, Rastas M. Prevention of type 2 diabetes mellitus by changes in lifestyle among subjects with impaired glucose tolerance. N Engl J Med. 2001;344(18):1343–50. 11333990 10.1056/NEJM200105033441801

[6] Abbasi A, Peelen LM, Corpeleijn E, van der Schouw YT, Stolk RP, Spijkerman AM, van der Moons AD, Navis KG, Bakker G. Prediction models for risk of developing type 2 diabetes: systematic literature search and independent external validation study. BMJ. 2012;345:e5900. 22990994 10.1136/bmj.e5900 PMC3445426

[7] Hippisley-Cox J, Coupland C. Development and validation of QDiabetes-2018 risk prediction algorithm to estimate future risk of type 2 diabetes: cohort study. BMJ. 2017;359:j5019. 29158232 10.1136/bmj.j5019 PMC5694979

[8] Langenberg C, Lotta LA. Genomic insights into the causes of type 2 diabetes. Lancet. 2018;391(10138):2463–74. 29916387 10.1016/S0140-6736(18)31132-2

[9] Merino J, Leong A, Liu C-T, Porneala B, Walford GA, von Grotthuss M, Wang TJ, Flannick J, Dupuis J, Levy D. Metabolomics insights into early type 2 diabetes pathogenesis and detection in individuals with normal fasting glucose. Diabetologia. 2018;61:1315–24. 29626220 10.1007/s00125-018-4599-x PMC5940516

[10] Carrasco-Zanini J, Pietzner M, Lindbohm JV, Wheeler E, Oerton E, Kerrison N, Simpson M, Westacott M, Drolet D, Kivimaki M. Proteomic signatures for identification of impaired glucose tolerance. Nat Med. 2022;28(11):2293–300. 36357677 10.1038/s41591-022-02055-z PMC7614638

[11] Suzuki K, Hatzikotoulas K, Southam L, Taylor HJ, Yin X, Lorenz KM, Mandla R, Huerta-Chagoya A, Melloni GEM, Kanoni S, et al. Genetic drivers of heterogeneity in type 2 diabetes pathophysiology. Nature. 2024;627(8003):347–57. 38374256 10.1038/s41586-024-07019-6 PMC10937372

[12] Roberts LD, Koulman A, Griffin JL. Towards metabolic biomarkers of insulin resistance and type 2 diabetes: progress from the metabolome. lancet Diabetes Endocrinol. 2014;2(1):65–75. 24622670 10.1016/S2213-8587(13)70143-8

[13] Chen ZZ, Gerszten RE. Metabolomics and proteomics in type 2 diabetes. Circ Res. 2020;126(11):1613–27. 32437301 10.1161/CIRCRESAHA.120.315898 PMC11118076

[14] Meigs JB, Shrader P, Sullivan LM, McAteer JB, Fox CS, Dupuis J, Manning AK, Florez JC, Wilson PW. D’Agostino Sr RB: Genotype score in addition to common risk factors for prediction of type 2 diabetes. N Engl J Med. 2008;359(21):2208–19. 19020323 10.1056/NEJMoa0804742 PMC2746946

[15] Talmud PJ, Cooper JA, Morris RW, Dudbridge F, Shah T, Engmann J, Dale C, White J, McLachlan S, Zabaneh D. Sixty-five common genetic variants and prediction of type 2 diabetes. Diabetes. 2015;64(5):1830–40. 25475436 10.2337/db14-1504 PMC4407866

[16] Morze J, Wittenbecher C, Schwingshackl L, Danielewicz A, Rynkiewicz A, Hu FB, Guasch-Ferré M. Metabolomics and type 2 diabetes risk: an updated systematic review and meta-analysis of prospective cohort studies. Diabetes Care. 2022;45(4):1013–24. 35349649 10.2337/dc21-1705 PMC9016744

[17] Kurgan N, Kjærgaard Larsen J, Deshmukh AS. Harnessing the power of proteomics in precision diabetes medicine. Diabetologia. 2024;67(5):783–97. 38345659 10.1007/s00125-024-06097-5

[18] Carrasco-Zanini J, Pietzner M, Wheeler E, Kerrison ND, Langenberg C, Wareham NJ. Multi-omic prediction of incident type 2 diabetes. Diabetologia. 2024;67(1):102–12. 37889320 10.1007/s00125-023-06027-x PMC10709231

[19] Xie R, Vlaski T, Trares K, Herder C, Holleczek B, Brenner H, Schöttker B. Large-scale proteomics improve risk prediction for type 2 diabetes. Diabetes Care 2025;48(6):922-926 . 10.2337/dc24-2478 40178901

[20] Xie R, Herder C, Sha S, Peng L, Brenner H, Schoettker B. Novel type 2 diabetes prediction score based on traditional risk factors and circulating metabolites: model derivation and validation in two large cohort studies. eClinicalMedicine 2025;79:102971. 10.1016/j.eclinm.2024.102971 PMC11667638 39720612

[21] Rahman M, Simmons RK, Harding AH, Wareham NJ, Griffin SJ. A simple risk score identifies individuals at high risk of developing Type 2 diabetes: a prospective cohort study. Fam Pract. 2008;25(3):191–6. 18515811 10.1093/fampra/cmn024

[22] Thompson DJ, Wells D, Selzam S, Peneva I, Moore R, Sharp K, Tarran WA, Beard EJ, Riveros-Mckay F, Giner-Delgado C, et al. A systematic evaluation of the performance and properties of the UK Biobank Polygenic Risk Score (PRS) Release. PLoS ONE. 2024;19(9):e0307270. 39292644 10.1371/journal.pone.0307270 PMC11410272

[23] Littlejohns TJ, Sudlow C, Allen NE, Collins R. UK Biobank: opportunities for cardiovascular research. Eur Heart J. 2019;40(14):1158–66. 28531320 10.1093/eurheartj/ehx254 PMC6451771

[24] Echouffo-Tcheugui JB, Selvin E. Prediabetes and what it means: the epidemiological evidence. Annu Rev Public Health. 2021;42:59–77. 33355476 10.1146/annurev-publhealth-090419-102644 PMC8026645

[25] Westerman KE, Miao J, Chasman DI, Florez JC, Chen H, Manning AK, Cole JB. Genome-wide gene-diet interaction analysis in the UK Biobank identifies novel effects on hemoglobin A1c. Hum Mol Genet. 2021;30(18):1773–83. 33864366 10.1093/hmg/ddab109 PMC8411984

[26] Sun BB, Chiou J, Traylor M, Benner C, Hsu Y-H, Richardson TG, Surendran P, Mahajan A, Robins C, Vasquez-Grinnell SG. Plasma proteomic associations with genetics and health in the UK Biobank. Nature. 2023;622(7982):329–38. 37794186 10.1038/s41586-023-06592-6 PMC10567551

[27] Wik L, Nordberg N, Broberg J, Björkesten J, Assarsson E, Henriksson S, Grundberg I, Pettersson E, Westerberg C, Liljeroth E. Proximity extension assay in combination with next-generation sequencing for high-throughput proteome-wide analysis. Mol Cell Proteom 2021;20:100168. 10.1016/j.mcpro.2021.100168 PMC8633680 34715355

[28] Assarsson E, Lundberg M, Holmquist G, Björkesten J, Bucht Thorsen S, Ekman D, Eriksson A, Rennel Dickens E, Ohlsson S, Edfeldt G. Homogenous 96-plex PEA immunoassay exhibiting high sensitivity, specificity, and excellent scalability. PLoS ONE. 2014;9(4):e95192. 24755770 10.1371/journal.pone.0095192 PMC3995906

[29] Lundberg M, Eriksson A, Tran B, Assarsson E, Fredriksson S. Homogeneous antibody-based proximity extension assays provide sensitive and specific detection of low-abundant proteins in human blood. Nucleic Acids Res. 2011;39(15):e102–102. 21646338 10.1093/nar/gkr424 PMC3159481

[30] Ritchie SC, Surendran P, Karthikeyan S, Lambert SA, Bolton T, Pennells L, Danesh J, Di Angelantonio E, Butterworth AS, Inouye M. Quality control and removal of technical variation of NMR metabolic biomarker data in ~ 120,000 UK Biobank participants. Sci data. 2023;10(1):64. 36720882 10.1038/s41597-023-01949-y PMC9887579

[31] Khera AV, Chaffin M, Aragam KG, Haas ME, Roselli C, Choi SH, Natarajan P, Lander ES, Lubitz SA, Ellinor PT, et al. Genome-wide polygenic scores for common diseases identify individuals with risk equivalent to monogenic mutations. Nat Genet. 2018;50(9):1219–24. 30104762 10.1038/s41588-018-0183-z PMC6128408

[32] Mars N, Koskela JT, Ripatti P, Kiiskinen TTJ, Havulinna AS, Lindbohm JV, Ahola-Olli A, Kurki M, Karjalainen J, Palta P, et al. Polygenic and clinical risk scores and their impact on age at onset and prediction of cardiometabolic diseases and common cancers. Nat Med. 2020;26(4):549–57. 32273609 10.1038/s41591-020-0800-0

[33] Bragg F, Trichia E, Aguilar-Ramirez D, Bešević J, Lewington S, Emberson J. Predictive value of circulating NMR metabolic biomarkers for type 2 diabetes risk in the UK Biobank study. BMC Med. 2022;20(1):159. 35501852 10.1186/s12916-022-02354-9 PMC9063288

[34] Stekhoven DJ, Bühlmann P. MissForest–non-parametric missing value imputation for mixed-type data. Bioinformatics. 2012;28(1):112–8. 22039212 10.1093/bioinformatics/btr597

[35] Kang L, Chen W, Petrick NA, Gallas BD. Comparing two correlated C indices with right-censored survival outcome: a one-shot nonparametric approach. Stat Med. 2015;34(4):685–703. 25399736 10.1002/sim.6370 PMC4314453

[36] Kerr KF, Wang Z, Janes H, McClelland RL, Psaty BM, Pepe MS. Net reclassification indices for evaluating risk-prediction instruments: a critical review. Epidemiol (Cambridge Mass). 2014;25(1):114. 10.1097/EDE.0000000000000018 PMC3918180 24240655

[37] Li L, Pang S, Starnecker F, Mueller-Myhsok B, Schunkert H. Integration of a polygenic score into guideline-recommended prediction of cardiovascular disease. Eur Heart J. 2024;45(20):1843–52. 38551411 10.1093/eurheartj/ehae048 PMC11129792

[38] Zanini JC, Pietzner M, Langenberg C. Integrating genetics and the plasma proteome to predict the risk of type 2 diabetes. Curr Diab Rep. 2020;20(11):60. 33033935 10.1007/s11892-020-01340-w PMC7543966

[39] Läll K, Mägi R, Morris A, Metspalu A, Fischer K. Personalized risk prediction for type 2 diabetes: the potential of genetic risk scores. Genet Med. 2017;19(3):322–9. 27513194 10.1038/gim.2016.103 PMC5506454

[40] Satheesh G, Ramachandran S, Jaleel A. Metabolomics-based prospective studies and prediction of type 2 diabetes mellitus risks. Metab Syndr Relat Disord. 2020;18(1):1–9. 31634052 10.1089/met.2019.0047

[41] Meigs JB. The Genetic epidemiology of type 2 diabetes: opportunities for health translation. Curr Diab Rep. 2019;19(8):62. 31332628 10.1007/s11892-019-1173-y PMC8059416

[42] Floegel A, Stefan N, Yu Z, Mühlenbruch K, Drogan D, Joost HG, Fritsche A, Häring HU, de Hrabě M, Peters A, et al. Identification of serum metabolites associated with risk of type 2 diabetes using a targeted metabolomic approach. Diabetes. 2013;62(2):639–48. 23043162 10.2337/db12-0495 PMC3554384

[43] Huth C, von Toerne C, Schederecker F, de Las Heras Gala T, Herder C, Kronenberg F, Meisinger C, Rathmann W, Koenig W, Waldenberger M, et al. Protein markers and risk of type 2 diabetes and prediabetes: a targeted proteomics approach in the KORA F4/FF4 study. Eur J Epidemiol. 2019;34(4):409–22. 30599058 10.1007/s10654-018-0475-8 PMC6451724

[44] Luo H, Bauer A, Nano J, Petrera A, Rathmann W, Herder C, Hauck SM, Sun BB, Hoyer A, Peters A, et al. Associations of plasma proteomics with type 2 diabetes and related traits: results from the longitudinal KORA S4/F4/FF4 Study. Diabetologia. 2023;66(9):1655–68. 37308750 10.1007/s00125-023-05943-2

[45] Rooney MR, Chen J, Echouffo-Tcheugui JB, Walker KA, Schlosser P, Surapaneni A, Tang O, Chen J, Ballantyne CM, Boerwinkle E, et al. Proteomic predictors of incident diabetes: results from the atherosclerosis risk in communities (ARIC) study. Diabetes Care. 2023;46(4):733–41. 36706097 10.2337/dc22-1830 PMC10090896

[46] Yao P, Iona A, Pozarickij A, Said S, Wright N, Lin K, Millwood I, Fry H, Kartsonaki C, Mazidi M. Proteomic analyses in diverse populations improved risk prediction and identified new drug targets for type 2 diabetes. Diabetes Care. 2024;47(6):1012–9. 38623619 10.2337/dc23-2145 PMC7615965

[47] Pencina MJ, D’Agostino Sr RB, D’Agostino Jr RB, Vasan RS. Evaluating the added predictive ability of a new marker: from area under the ROC curve to reclassification and beyond. Stat Med. 2008;27(2):157–72. 17569110 10.1002/sim.2929

[48] Tiret L, Godefroy T, Lubos E, Nicaud V, Tregouet DA, Barbaux S, Schnabel R, Bickel C, Espinola-Klein C, Poirier O, et al. Genetic analysis of the interleukin-18 system highlights the role of the interleukin-18 gene in cardiovascular disease. Circulation. 2005;112(5):643–50. 16043644 10.1161/CIRCULATIONAHA.104.519702

[49] Herder C, de Las Heras Gala T, Carstensen-Kirberg M, Huth C, Zierer A, Wahl S, Sudduth-Klinger J, Kuulasmaa K, Peretz D, Ligthart S, et al. Circulating levels of interleukin 1-receptor antagonist and risk of cardiovascular disease: meta-analysis of six population-based cohorts. Arterioscler Thromb Vasc Biol. 2017;37(6):1222–7. 28428221 10.1161/ATVBAHA.117.309307

[50] Mallat Z, Corbaz A, Scoazec A, Besnard S, Lesèche G, Chvatchko Y, Tedgui A. Expression of interleukin-18 in human atherosclerotic plaques and relation to plaque instability. Circulation. 2001;104(14):1598–603. 11581135 10.1161/hc3901.096721

[51] Ferraro RA, Ogunmoroti O, Zhao D, Ndumele CE, Rao V, Pandey A, Larson NB, Bielinski SJ, Michos ED. Hepatocyte growth factor and incident heart failure subtypes: the multi-ethnic study of atherosclerosis (MESA). J Card Fail. 2021;27(9):981–90. 34051347 10.1016/j.cardfail.2021.04.022 PMC8434952

[52] Balling M, Afzal S, Davey Smith G, Varbo A, Langsted A, Kamstrup PR, Nordestgaard BG. Elevated LDL triglycerides and atherosclerotic Risk. J Am Coll Cardiol. 2023;81(2):136–52. 36631208 10.1016/j.jacc.2022.10.019

[53] Hansen M, Walmod PS. IGSF9 family proteins. Neurochem Res. 2013;38(6):1236–51. 23417431 10.1007/s11064-013-0999-y

[54] van Eekeren LE, de Mast Q, Meeder EM, Navas A, Groenendijk AL, Blaauw MJ, Vos WA, Vadaq N, Dos Santos JC, Rutten J. Plasma proteomic signatures of liver steatosis and fibrosis in people living with HIV: a cross-sectional study. EBioMedicine 2024;109:105407. 10.1016/j.ebiom.2024.105407 PMC11513669 39426127

[55] Singh A, Ganslmeier M, Tutino M, Park Y-C, Machann J, Schick F, Peter A, Lehmann R, Wang Y, Cheng Y. Longitudinal proteogenomic analysis reveals mechanistic insights into the progression from prediabetes to type 2 diabetes. medRxiv 2026 Feb 16. 10.64898/2026.02.13.26346161

[56] Jiang Q, Li J, Wu S, Zhang L, Dong L, Weng S, Tang W, Chen S. Enterococcus faecalis impairs IGSF9-dependent C1q degradation to accelerate MAFLD-HCC progression. Gut Microbes Rep. 2026;3(1):2600896. 41907518 10.1080/29933935.2025.2600896 PMC12938874

[57] Li Y, Deng Y, Zhao Y, Zhang W, Zhang S, Zhang L, Wang B, Xu Y, Chen S. Immunoglobulin superfamily 9 (IGSF9) is trans-activated by p53, inhibits breast cancer metastasis via FAK. Oncogene. 2022;41(41):4658–72. 36088502 10.1038/s41388-022-02459-8 PMC9546770

[58] Sarwar N, Gao P, Seshasai SR, Gobin R, Kaptoge S, Di Angelantonio E, Ingelsson E, Lawlor DA, Selvin E, Stampfer M, et al. Diabetes mellitus, fasting blood glucose concentration, and risk of vascular disease: a collaborative meta-analysis of 102 prospective studies. Lancet. 2010;375(9733):2215–22. 20609967 10.1016/S0140-6736(10)60484-9 PMC2904878

[59] Carrasco-Zanini J, Pietzner M, Davitte J, Surendran P, Croteau-Chonka DC, Robins C, Torralbo A, Tomlinson C, Grünschläger F, Fitzpatrick N, et al. Proteomic signatures improve risk prediction for common and rare diseases. Nat Med. 2024;30(9):2489–98. 39039249 10.1038/s41591-024-03142-z PMC11405273

[60] Carrasco-Zanini J, Pietzner M, Koprulu M, Wheeler E, Kerrison ND, Wareham NJ, Langenberg C. Proteomic prediction of diverse incident diseases: a machine learning-guided biomarker discovery study using data from a prospective cohort study. Lancet Digit Health. 2024;6(7):e470–9. 38906612 10.1016/S2589-7500(24)00087-6

