# Supplementary material for: Large-scale multi-omics enhance risk prediction for type 2 diabetes
Source: Cardiovasc Diabetol. 2026 May 28;25:166. doi: 10.1186/s12933-026-03223-y (PMC13217982; doi:10.1186/s12933-026-03223-y)
Supplement: Supplementary file 1 — Supplementary Material 1. [file 12933_2026_3223_MOESM1_ESM.docx]

**Supplemental Materials to**

**Large-Scale Multi-Omics Enhance Risk Prediction for Type 2 Diabetes**

Table of Contents

[Supplemental Table S1. Selected multi-omic biomarkers and data sources 2](#_Toc7060)

[Supplemental Table S2. Diagnosis and medication codes used for ascertainment of incident type 2 diabetes in the UK Biobank 3](#_Toc12484)

[Supplemental Table S3. ß-coefficients of the different extensions of the clinical CDRS model with multi-omics data for 10-year prediction of type 2 diabetes 5](#_Toc18538)

[Supplemental Figure S1. Flowchart of participant selection 7](#_Toc3398)

[Supplemental Figure S2. Calibration curves of the clinical CDRS model and its multi-omics extensions for 10-year type 2 diabetes risk prediction in the validation set (N = 19,732) 8](#_Toc9973)

[Supplemental Figure S3. Subgroup analyses of C-index (95% CI) for type 2 diabetes risk prediction models stratified by age group, BMI category, pre-diabetes status, and sex 9](#_Toc15889)

[References to Supplemental Materials 10](#_Toc13684)

**Supplemental Table S1.** Selected multi-omic biomarkers and data sources

| **Omics** | **Biomarkers** | **Reference** |
| --- | --- | --- |
| PRS | Standard PRS for type 2 diabetes | The standard T2D-PRS was derived using a genome-wide meta-analysis of 5 external GWAS datasets comprising 1,349,223 individuals and has been previously published [1]. |
| Metabolites | 3-hydroxybutyrate, acetate, citrate, glutamine, glucose, IDL-CE-pct, LA-pct, lactate, M-LDL-TG-pct, pyruvate, tyrosine | The metabolites were selected using bootstrap-LASSO regression in the UKB (n=60,362). See previous work [2]. |
| Proteins | ADAMTSL2, ADGRG1, ADIPOQ, APOM, CDH2, HGF, IGFBP1, IGSF3, IGSF9, IL18R1, IL1RN, LGALS4, PON3, RTN4R, TNFSF12 | The proteins were selected using bootstrap-LASSO regression in the UKB (n=14,759). See previous work [3]. |

Metabolites were quantified from EDTA plasma using nuclear magnetic resonance spectroscopy, and proteins were measured from EDTA plasma using Olink proximity extension assays.

**Abbreviations:** ADAMTSL2, A disintegrin and metalloproteinase with thrombospondin repeats–like 2; ADGRG1, Adhesion G-protein coupled receptor G1; CDH2, Cadherin-2; CDRS, Cambridge Diabetes Risk Score; HGF, Hepatocyte growth factor; IDL-CE-pct, cholesteryl esters to total lipids in IDL percentage; IGFBP1, Insulin-like growth factor-binding protein 1; IGSF, Immunoglobulin superfamily member; IL1RN, Interleukin-1 receptor antagonist ; IL18R1, Interleukin-18 receptor 1; LA-pct, linoleic acid to total fatty acids percentage; M-LDL-TG-pct, triglycerides to total lipids in medium LDL percentage; PON3, paraoxonase/lactonase 3; PRS, polygenic risk score; RTN4R, Reticulon-4 receptor; T2D, type 2 diabetes; T2D-PRS, polygenic risk score for type 2 diabetes; TNFSF12, TNF-related weak inducer of apoptosis.

**Supplemental Table S2.** Diagnosis and medication codes used for ascertainment of incident type 2 diabetes in the UK Biobank

| **Data source** | **Code format** | **Codes** |
| --- | --- | --- |
| ***Diagnosis codes*** | | |
| Hospital inpatient data (Hospital Episode Statistics) | ICD-10 | E11 (Type 2 diabetes mellitus), E11.0–E11.9 (all subcodes) |
| Death registry | ICD-10 | E11 (Type 2 diabetes mellitus), E11.0–E11.9 (all subcodes) |
| Primary care (General Practice records) | Read v2 | C1001, C1011, C1021, C1031, C1041, C1051, C1061, C1071, C1074, C109., C1090–C1099, C109A–C109K, C10F., C10F0–C10F9, C10FA–C10FR, C10P1, C10y1, C10z1, L1806, L180B |
| Primary care (General Practice records) | Read CTV3 | 66A3., C1001, C1011, C1021, C1031, C1041, C1051, C1061, C1071, C1074, C109., C1090–C1097, C10y1, C10z1, L1806, X40J6, XaagF, XacsX, XacsY, XaELQ, XaEnp, XaEn9, XaF05, XaFm4, XaFn7, XaFn8, XaFn9, XaFWi, XaIfG, XaIfl, XaIzQ, XaIzR, XaJQp, XaKyX, XaXZR, XE10F, XE12A, XM19j |
| Self-report (UK Biobank verbal interview) | UKB coding | Data-Field 20002, coding 1223 (type 2 diabetes) |
| ***Glucose-lowering medication codes*** | | |
| Primary care (GP prescription records) | BNF chapter 6.1.1 (Insulins) | BNF codes: 060101xxxx Including all insulin preparations (human insulins, insulin analogues) |
| Primary care (GP prescription records) | BNF chapter 6.1.2 (Antidiabetic drugs) | BNF codes: 060102xxxx Including: biguanides (metformin), sulfonylureas (glibenclamide, gliclazide, glimepiride, glipizide, tolbutamide), alpha-glucosidase inhibitors (acarbose), thiazolidinediones (pioglitazone), DPP-4 inhibitors (sitagliptin, vildagliptin, saxagliptin, linagliptin, alogliptin), GLP-1 receptor agonists (exenatide, liraglutide, lixisenatide, dulaglutide, semaglutide), SGLT2 inhibitors (dapagliflozin, canagliflozin, empagliflozin), meglitinides (repaglinide, nateglinide) |
| Primary care (GP prescription records) | Read v2 / CTV3 | All codes under the hierarchy f1... (Antidiabetic drugs) Including: f11.. (Insulins), f12.. (Sulfonylureas), f13.. (Biguanides), f14.. (Other antidiabetics), and all sub-codes |
| Self-report (UK Biobank verbal interview) | UKB coding | Data-Field 20003 (Treatment/medication code) Codes: 1140857494 (metformin), 1140874646 (gliclazide), 1140874674 (glimepiride), 1140874744 (pioglitazone), 1140868902 (insulin product), and all other glucose-lowering medication codes from UKB coding 4 |
| ***Exclusion codes for other diabetes types*** | | |
| Hospital inpatient data / Death registry | ICD-10 | E10 (Type 1 diabetes mellitus), E12 (Malnutrition-related diabetes mellitus), E13 (Other specified diabetes mellitus), E14 (Unspecified diabetes mellitus), O24 (Diabetes mellitus in pregnancy) |
| Primary care | Read v2 / CTV3 | Codes corresponding to type 1 diabetes, gestational diabetes, and other specified diabetes types |
| Self-report | UKB coding | Data-Field 20002, coding 1222 (type 1 diabetes); Data-Field 20002, coding 1521 (gestational diabetes) |

**Abbreviations:** BNF, British National Formulary; CTV3, Clinical Terms Version 3; DPP-4, dipeptidyl peptidase-4; GLP-1, glucagon-like peptide-1; GP, general practitioner; ICD-10, International Classification of Diseases, 10th Revision; SGLT2, sodium-glucose co-transporter 2; UKB, UK Biobank.

**Supplemental** **Table S3.** ß-coefficients of the different extensions of the clinical CDRS model with multi-omics data for 10-year prediction of type 2 diabetes

| **Variables** | **ß-coefficient** | |
| --- | --- | --- |
|  | **Clinical CDRS + Proteomics** | **Clinical CDRS + Multi-omics** |
| **Clinical CDRS** |  |  |
| Female | 0.2969 | 0.2796 |
| Prescribed anti-hypertension medication | 0.1220 | 0.1037 |
| Prescribed steroids | 0.7921 | 0.8197 |
| Age (per years) | 0.1518 | 0.1513 |
| Body mass index <25 kg/m^2^ | Ref | Ref |
| Body mass index 25-27.49 kg/m^2^ | -0.1200 | -0.1289 |
| Body mass index 27.5-29.99 kg/m^2^ | 0.1371 | 0.0724 |
| Body mass index ≥ 30 kg/m^2^ | 0.3483 | 0.3420 |
| No first degree relative with diabetes | Ref | Ref |
| Parent or sibling with diabetes | 0.2367 | 0.2420 |
| Parent and sibling with diabetes | 0.4007 | 0.3416 |
| None-smoker | Ref | Ref |
| Ex-smoker | 0.1219 | 0.1232 |
| Current smoker | 0.1057 | 0.0733 |
| HbA_1c_ (per %) | 0.9569 | 0.8731 |
| **Added proteins (per 1 SD)** |  |  |
| ADAMTSL2 | 0.0464 | 0.0470 |
| ADGRG1 | -0.0051 | 0.0021 |
| ADIPOQ | -0.0887 | -0.0721 |
| APOM | -0.1501 | -0.1454 |
| CDH2 | 0.1258 | 0.0998 |
| HGF | 0.0982 | 0.1604 |
| IGFBP1 | -0.0924 | -0.1202 |
| IGSF3 | 0.0068 | 0.0164 |
| IGSF9 | 0.1194 | 0.0719 |
| IL18R1 | 0.1870 | 0.1475 |
| IL1RN | 0.0451 | 0.0595 |
| LGALS4 | 0.0961 | 0.0640 |
| PON3 | -0.0730 | -0.0466 |
| RTN4R | 0.0691 | 0.0789 |
| TNFSF12 | -0.1194 | -0.1296 |
| **Added metabolites** **(per 1 SD)** |  |  |
| 3-hydroxybutyrate | – | 0.0362 |
| Acetate | – | 0.0168 |
| Citrate | – | 0.0187 |
| Glutamine | – | 0.0740 |
| Glucose | – | 0.1901 |
| IDL-CE-pct | – | -0.0070 |
| LA-pct | – | -0.0740 |
| Lactate | – | -0.0052 |
| M-LDL-TG-pct | – | 0.0879 |
| Pyruvate | – | -0.0468 |
| Tyrosine | – | -0.1105 |
| **Added PRS** **(per 1 SD)** |  |  |
| T2D-PRS | – | 0.2964 |

**Abbreviations:** ADAMTSL2, A disintegrin and metalloproteinase with thrombospondin repeats-like 2; ADGRG1, Adhesion G-protein coupled receptor G1; ADIPOQ, Adiponectin; APOM, Apolipoprotein M; CDH2, Cadherin-2; CDRS, Cambridge Diabetes Risk Score; HGF, Hepatocyte growth factor; IDL-CE-pct, cholesteryl esters to total lipids in IDL percentage; IGFBP1, Insulin-like growth factor-binding protein 1; IGSF, Immunoglobulin superfamily member; IL1RN, Interleukin-1 receptor antagonist; IL18R1, Interleukin-18 receptor 1; LA-pct, linoleic acid to total fatty acids percentage; M-LDL-TG-pct, triglycerides to total lipids in medium LDL percentage; PON3, paraoxonase/lactonase 3; PRS, polygenic risk score; Ref, reference; RTN4R, Reticulon-4 receptor; SD, standard deviation; T2D, type 2 diabetes; TNFSF12, TNF-related weak inducer of apoptosis.


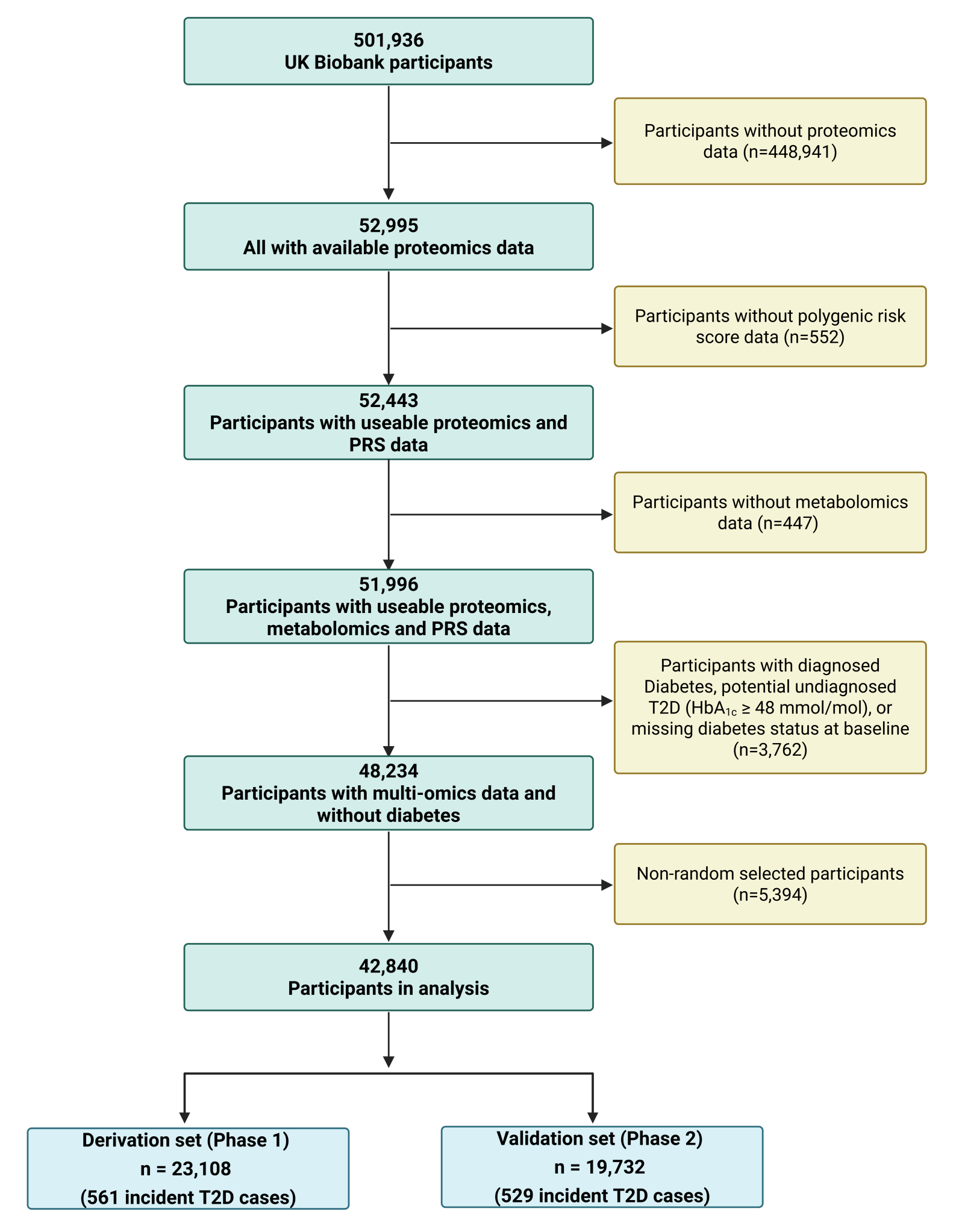


**Supplemental Figure S1.** Flowchart of participant selection

**Abbreviations:** HbA_1c_, glycated hemoglobin; PRS, polygenic risk score; T2D, type 2 diabetes.

**
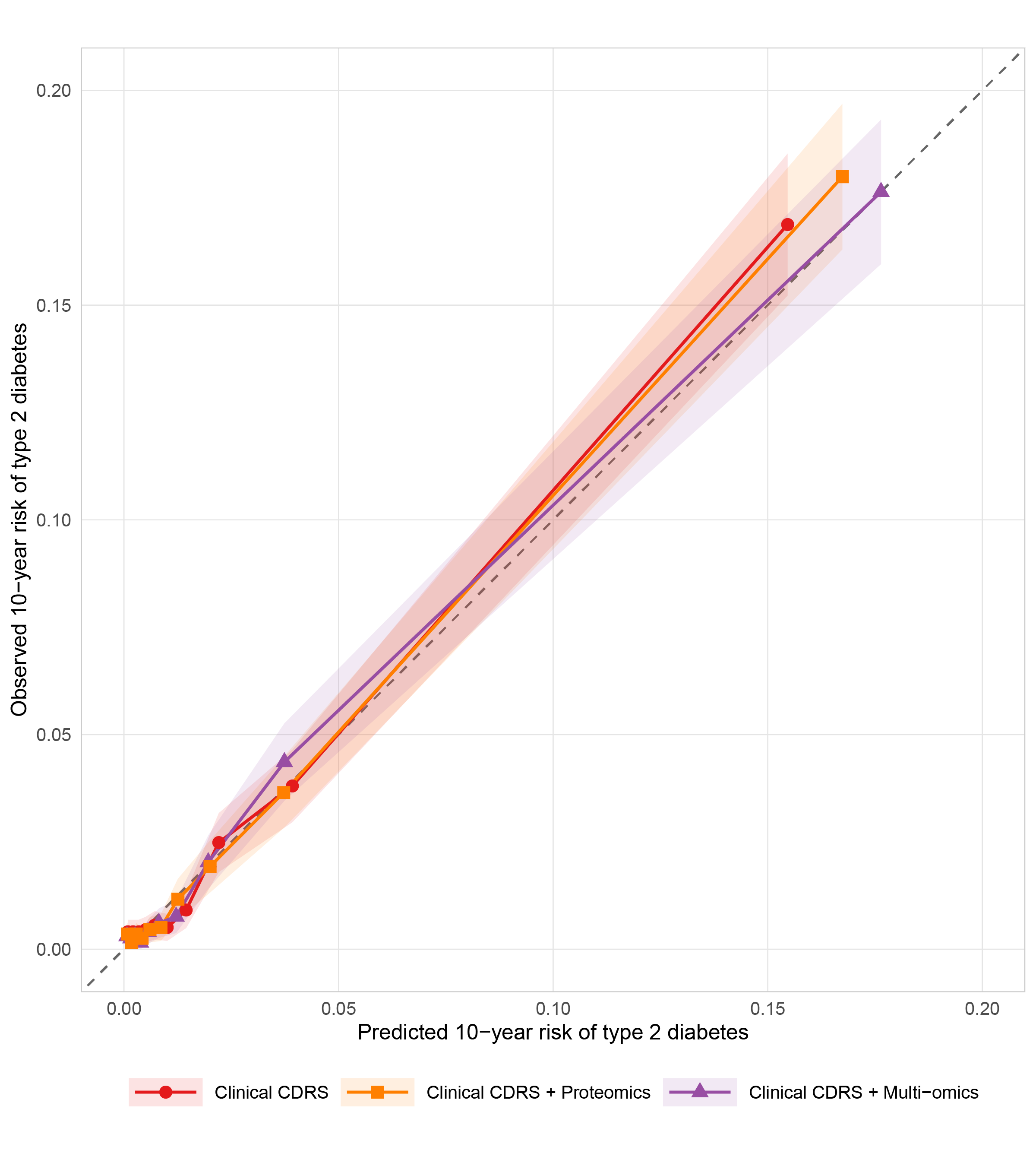
**

**Supplemental Figure S2.** Calibration curves of the clinical CDRS model and its multi-omics extensions for 10-year type 2 diabetes risk prediction in the validation set (N = 19,732)

Predicted 10-year absolute risks were computed using the Breslow estimator of the baseline survival function from models fitted in the derivation set. Participants in the validation set were grouped into deciles of predicted risk, and observed event rates were plotted against mean predicted risks within each decile. Shaded areas represent 95% confidence intervals based on the binomial distribution. The dashed diagonal line indicates perfect calibration. The multi-omics model refers to the integration of proteomics, metabolomics, and the T2D-PRS.

**Abbreviations:** CDRS, Cambridge Diabetes Risk Score; T2D-PRS, polygenic risk score for type 2 diabetes.

**
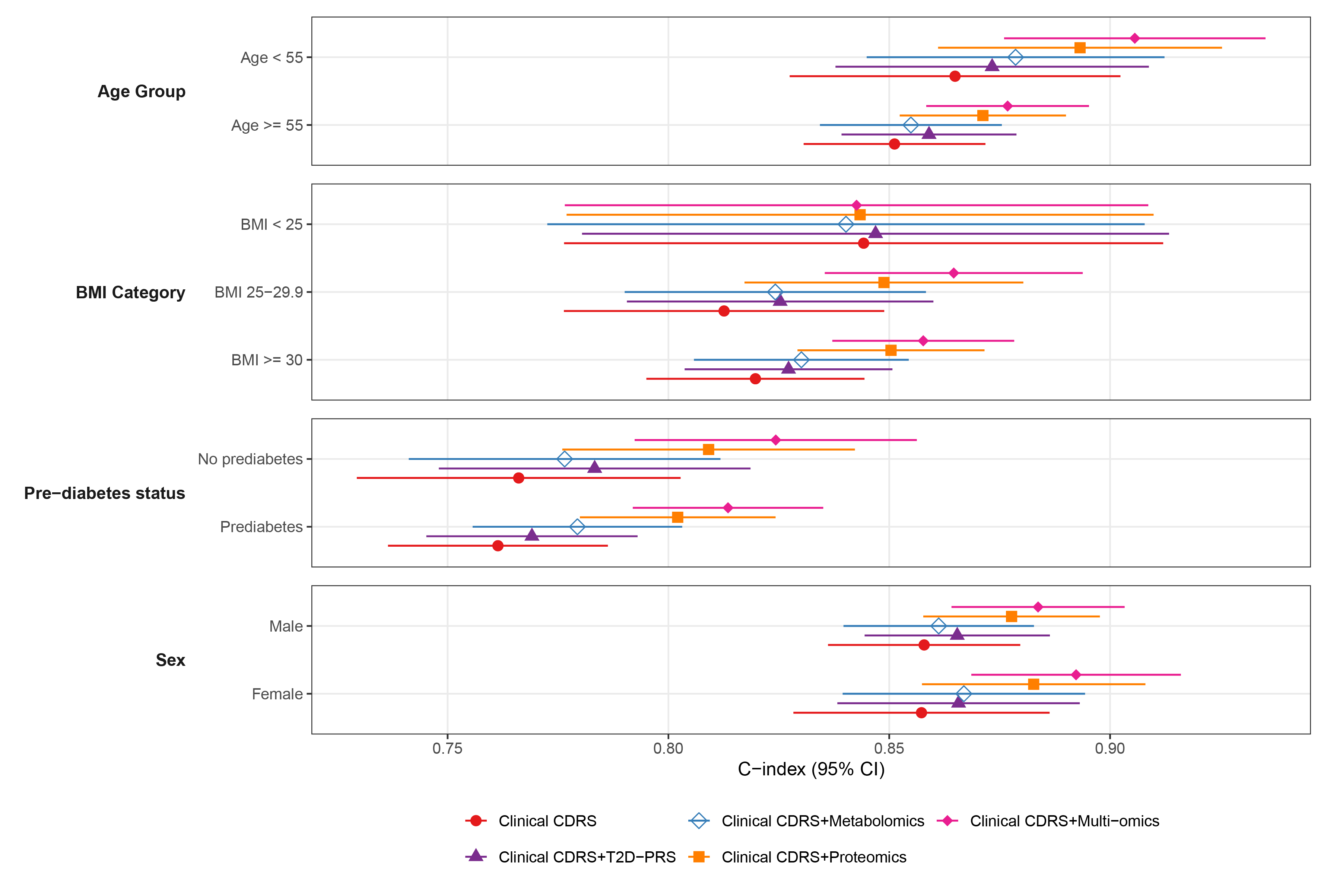
**

**Supplemental Figure S3.** Subgroup analyses of C-index (95% CI) for type 2 diabetes risk prediction models stratified by age group, BMI category, pre-diabetes status, and sex

Five models are shown: Clinical CDRS (red circle), Clinical CDRS+T2D-PRS (purple triangle), Clinical CDRS+Metabolomics (blue diamond), Clinical CDRS+Proteomics (orange square), and Clinical CDRS+Multi-omics (pink diamond). All models were evaluated in the validation set.

**Abbreviations:** BMI, body mass index; CDRS, Cambridge Diabetes Risk Score; CI, confidence interval; T2D-PRS, polygenic risk score for type 2 diabetes.

**References to Supplemental Materials**

1. Thompson DJ, Wells D, Selzam S, Peneva I, Moore R, Sharp K, Tarran WA, Beard EJ, Riveros-Mckay F, Giner-Delgado C *et al*: **A systematic evaluation of the performance and properties of the UK Biobank Polygenic Risk Score (PRS) Release**. *PLoS One* 2024, **19**(9):e0307270.

2. Xie R, Herder C, Sha S, Peng L, Brenner H, Schoettker B: **Novel type 2 diabetes prediction score based on traditional risk factors and circulating metabolites: Model derivation and validation in two large cohort studies**. *eClinicalMedicine* 2025, **79**.

3. Xie R, Vlaski T, Trares K, Herder C, Holleczek B, Brenner H, Schöttker B: **Large-Scale Proteomics Improve Risk Prediction for Type 2 Diabetes**. *Diabetes Care* 2025.
